# Clinical effect of immunomodulatory therapy in periodontitis: a systematic review and meta-analysis

**DOI:** 10.3389/fbioe.2025.1693365

**Published:** 2025-11-20

**Authors:** Yubing Zhang, Xu Qin, Jiexuan Yang, Hannah M. Rogers, Babak Baban, Siwei Tian

**Affiliations:** 1 School of Stomatology, Tongji Medical College, Huazhong University of Science and Technology, Wuhan, China; 2 Hospital of Stomatology, Guanghua School of Stomatology, Sun Yat-sen University, Guangzhou, China; 3 Department of Stomatology, Tongji Hospital, Tongji Medical College, Huazhong University of Science and Technology, Wuhan, China; 4 Hubei Province Key Laboratory of Oral and Maxillofacial Development and Regeneration, Wuhan, China; 5 Department of Oral Biology and Diagnostic Services, Dental College of Georgia, Augusta University, Augusta, GA, United States

**Keywords:** periodontitis, clinical trials, immunomodulation, immunotherapy, systematic review

## Abstract

**Objectives:**

To evaluate the clinical effect of immunomodulatory therapy in periodontitis, and to identify the possible key inflammatory factors to intervene to modulate the immune status and improve periodontal conditions.

**Materials and methods:**

An electronic search was conducted for human-based studies published until October 2025 on MEDLINE (PubMed), ISI Web of Science, EMBASE, and the Cochrane Database. Randomized controlled trials (RCTs) comparing the effectiveness of immunotherapy and placebo were included. We also compared cytokine levels between the immunotherapy group and the non-immunotherapy group to identify the specific inflammatory mediators influenced by immunotherapy but not by SRP (Scaling and Root Planning). Meta-analyses with fixed and random effects models were performed. Risk of bias assessment was also performed for randomized controlled trials.

**Results:**

Of the 34 articles selected, 22 were included in the meta-analysis (n = 991). It was found that immunomodulatory therapy improved clinical attachment level (CAL), bleeding from probing (BOP), and depth of probing (PD) in patients with periodontitis. A three-group meta-analysis showed that immunotherapy affected periodontal disease progression by modulating local immune factors IL-1β, IL-17, IL-6, IL-8 and TNF-α, thus providing a potential statistically significant benefit.

**Conclusion:**

Immunotherapy influenced periodontal disease progression through the modulation of local immune factors. The data support the use of immunotherapy as an adjunct to conventional mechanical therapy. Further investigations are warranted to elucidate sources of heterogeneity of the results and examine the potentiality of using inflammatory cytokines as novel targets for the treatment of periodontal disease.

## 1 Introduction

Periodontitis is a common chronic multifactorial inflammatory disease (Jepsen et al., 2000; Basic and Dahlen, 2023). It is estimated that between 2011 and 2020, periodontitis in dentate adults was estimated to be around 62%, and severe periodontitis 23.6% (Kassebaum et al., 2014; Trindade et al., 2023). And with the rapid growth of the elderly population, periodontal health is becoming increasingly important (Eke et al., 2000). The etiology and mechanism of periodontitis is extremely complex. Periodontitis initiation and progression are related to multiple etiologic and risk factors. However, the most critical in periodontal disease pathogenesis is a reciprocally reinforced interplay between microbial dysbiosis and destructive inflammation. The occurrence and development of periodontitis is the result of the interaction between bacteria and the host. The aim of the treatment of periodontitis is to re-establish and maintain periodontal health and function by limiting the inflammatory process. Currently, the main treatment is mechanical debridement to remove calculus and plaque combined with anti-inflammatory therapy (Slots, 2000a; Albandar, 2000). However, the treatment result is not always satisfactory and stable maintenance (Graziani et al., 2000), periodontitis continues to break down periodontal apparatus and leads to tooth loss in some patients (Grover et al., 2016). How to promote the intrinsic repairing power of the local compromised tissue and re-establish the balance of inflammatory breakdown and regeneration is a challenging issue for both researchers and clinicians.

Local plaque and other stimulating factors affect periodontal homeostasis, and the local immune microenvironment changes (Hajishengallis et al., 2000; Mysak et al., 2014; Demkovych et al., 2023). The dysregulation of local homeostasis is mainly manifested by the intensification of pro-inflammatory processes and the inhibition of repair and regeneration processes. This dysbiosis eventually leads to destructive inflammation and bone resorption. Therefore, the progression of periodontitis can be divided into sequential stages of which periodontitis featuring advanced lesion bone loss (Qin et al., 2017a). Each stage has different immunological characteristics, including distinct distributions of immune cells and cytokines (Qin et al., 2017b; Bostanci et al., 2017). For example, inflammatory stimulation causes local periodontal Th17 cell infiltration and increased IL-17 secretion. After removal of the influence of external factors, implementing immunomodulatory interventions according to the characteristics of the immune microenvironment at different stages may help to maintain tissue homeostasis.

Recently, the applications of immunotherapies in the treatment of periodontitis have been noticed.

Immunotherapy is a treatment method that activates the body’s own immune system through various means to defend against diseases. These methods, including stem cell therapy, targeted drug therapy, microbial therapy, gene therapy, and other therapies, generally used as adjuvant therapy for classical mechanical debridement (Hajishengallis et al., 2000; Nile et al., 2016). For example, Omega-3 fatty acids adjuvant therapy can improve periodontal outcomes, not only by reducing inflammation, but also by limiting bone resorption and antibacterial effects (Chee et al., 2016; Azuma et al., 2022). Although various immune agents have been reported for the treatment of periodontitis, the results were not in exact agreement. Generally, the intervention of the immune factors can further facilitate the effectiveness of classic periodontal therapies, however the specific responding markers and the potential mechanism of immune regulation are still unclear.

In this study, clinical data were analyzed to find the potential targeting cytokines that characterize the local immune microenvironment in periodontitis. A systematic review and meta-analysis of all published clinical data was carried out to illustrate the function and work path of immunomodulatory therapy in the treatment of periodontitis for human application.

## 2 Materials and methods

The review protocol was specified before the implement of the study and registered in an international database (PROSPERO, registration number CRD42023413355). The protocol was compliant with the Cochrane Handbook (Higgins et al., 2019) and the results were presented following the instructions of the Preferred Reporting Items for Systematic Review and Meta-analysis (PRISMA) statement (Page et al., 2021).

### 2.1 Population, intervention, comparison, outcome (PICO) question

PICO: In human subjects with any form of periodontitis, does immunotherapy increase the clinical efficacy of periodontal treatment (P: humans with periodontitis; I: immunotherapy; C: not undergoing immunotherapy; O: clinical outcomes (probing depth (PD); clinical attachment level (CAL); bleeding on probing (BOP)) and immunological indices (relevant cytokines’ levels).

### 2.2 Search strategy

MeSH terms and Boolean operators see Supplementary Material 5 for complete search strategy.

The following electronic databases were searched for pertinent papers: EMBASE, MEDLINE/PubMed, Web of Science, and the Cochrane Database (including the Central Register of Controlled Trials (CENTER)) using a search strategy presented in Appendix 1. A manual search of the lists of the included references and of the table of contents (since 1990) of the Journal of Clinical Periodontology, Journal of Periodontal Research, Journal of Periodontology, Journal of Dental Research, Periodontal 2000 and Journal of Dentistry was also performed. Grey literature was searched interrogating OpenGrey and Greylit. ClinicalTrials.gov and the World Health Organization International Clinical Trials Registry Platform were also evaluated to explore ongoing or completed RCTs meeting the inclusion and exclusion criteria. The ambiguous or incomplete data were obtained by contacting the corresponding researchers. Only manuscripts in English were considered. Conference abstracts were excluded. The last electronic search was performed on 15 October 2025.

### 2.3 Study selection

Two reviewers (SW.T. and X.Q.) screened all titles and abstracts to remove duplicates. The full texts were further obtained and screened when studies were deemed eligible. Disagreements were resolved by discussion with a third reviewer (B.B.) to achieve a consensus.

### 2.4 Selection criteria

- Types of studies: randomized controlled clinical trials with at least a 2-month follow-up calculated from the beginning of the treatment protocol. A shorter follow-up was not considered as it would be unlike to reflect a meaningful difference in treatment response between test and control groups.
- Participants Types: 1) Studies included more than 10 adults (older than 18-year-old) patients diagnosed with periodontitis; 2) The patients affected with periodontitis were either systemically healthy or systemically compromised.
- Intervention Types: Test group: with immunotherapy (IgY against P. gingivalis gingipains, hyaluronan gels, melatonin, photodynamic therapy (PDT), probiotics, sub-antimicrobial dose of doxycycline, OZONE, unsaturated fatty acids, etc.). Control group: without immunotherapy.
- Outcomes: 1) Primary outcome: probing depth (PD), identified as the distance between the gingival margin and the periodontal pocket base. 2) Secondary outcomes: ①quantity of inflammatory biomarkers; ②clinical attachment level (CAL), defined as the distance from the cementoenamel junction to the tip of the periodontal probe; and ③ bleeding on probing (BOP). To be as inclusive as possible, the meta-analysis included data available for the closest time point up to 3 months (Martin-Cabezas et al., 2016).

### 2.5 Data extraction

Two authors (YB.Z. and X.Q.) independently extracted data. Any disagreements were resolved by discussion with a third reviewer (B.B.) until a consensus was reached. A standardized template developed from the Cochrane Collaboration was used to conduct data extraction. Overall, all the following information was extracted: 1) general information of the studies (including the authors names, year of publication, the region/country where the study was conducted, study period, and study design); 2) characteristics of participants (including ① the total participants’ numbers, age, gender, and numbers of included teeth or sites, ② the included periodontal disease stage and periodontal status inclusion criteria, ③ systemic conditions of the participants (including but not limited to smoking habit, systemic diseases, and long term medication situation), ④ the study groups (treatment of study and control groups \ number of participants or sites per group), and ⑤ outcome measures); 3) treatment modalities (including intervention measures, use of a placebo, and other relevant procedures (pre-treatment administrations and oral hygiene instructions, scaling and root planning (SRP) (Cobb, 2002), and supportive periodontal therapy (SPT) (Manresa et al., 2018))); 4) outcomes of the studies (at baseline, regular follow-up, and the end-of-trial).

In case of missing or blur information, attempts were made to contact the first or corresponding authors. Data was excluded from calculation until definite clarification was available. When the results of a study were published along its follow-up periods, only the longest follow-up was included. If a study was comparing more than two arms, the data from the test group was extracted for meta-analysis (Ramanauskaite et al., 2021).

### 2.6 Risk of bias (RoB) assessment

The assessment of methodological quality and risk of bias of each included research was conducted in duplicate (YB.Z. and SW.T.). The criteria for risk of bias evaluation were derived from the Cochrane Handbook for Systematic Reviews (Higgins et al., 2019). Each study was judged as at different level of bias (low, moderate, high, or unclear risk) based on the following aspects: 1) sequence generation (selection bias); 2) allocation concealment (selection bias); 3) masking of participants and personnel (performance bias); 4) blinding of outcome assessment (detection bias); 5) incomplete outcome data (attrition bias); 6) selective outcome reporting (reporting bias); and 7) other bias. The risk of bias was categorized as follows:

Low risk of bias (plausible bias unlikely to seriously alter the results) if all domains were at low risk of bias;

Unclear risk of bias (plausible bias that raises some doubt about the results) if one or more domains were at unclear risk of bias;

High risk of bias (plausible bias that seriously weakens confidence in the results) if ≥ 1 domains were at high risk of bias.

The RoB assessment results were presented graphically using Review Manager (Version 5.4 The Cochrane Collaboration).

### 2.7 Data synthesis

Differences between the immunotherapy and control groups were shown as weighted mean differences (WMD) and 95% confidence interval (CI) for continuous data. Mean differences and standard errors (SD) were inputted for each study. Forest plots were generated to represent WMD and 95% CI of primary and secondary outcomes from all included studies. The number of patients was identified as the unit of analysis. Heterogeneity ranged between 0% and 100% was assessed with χ^2^ test and I^2^ test with the lower values representing less heterogeneity. The analyses were performed using Review Manager 5.4 and reported adhered to the Preferred Reporting Items for Systematic Review and Meta-analyses (PRISMA) statement (Moher et al., 2009).

### 2.8 Assessment of heterogeneity

Statistical heterogeneity of the included studies was assessed with Cochran’s test with a significance threshold of p < 0.1. The quantification of the heterogeneity was calculated with I^2^ statistic. It represents the percentage of variation attributable to statistical heterogeneity and is categorized as low (25%–50%), moderate (51%–75%), or high (>75%) (Higgins et al., 2003).

### 2.9 Assessment of reporting biases

Small-study effects were assessed by testing for funnel plot asymmetry and by calculating Egger’s bias, for publication bias (Higgins et al., 2019). If asymmetry was evident, this was investigated and the possible causes were described.

## 3 Results

### 3.1 Study selection

**FIGURE 1:**
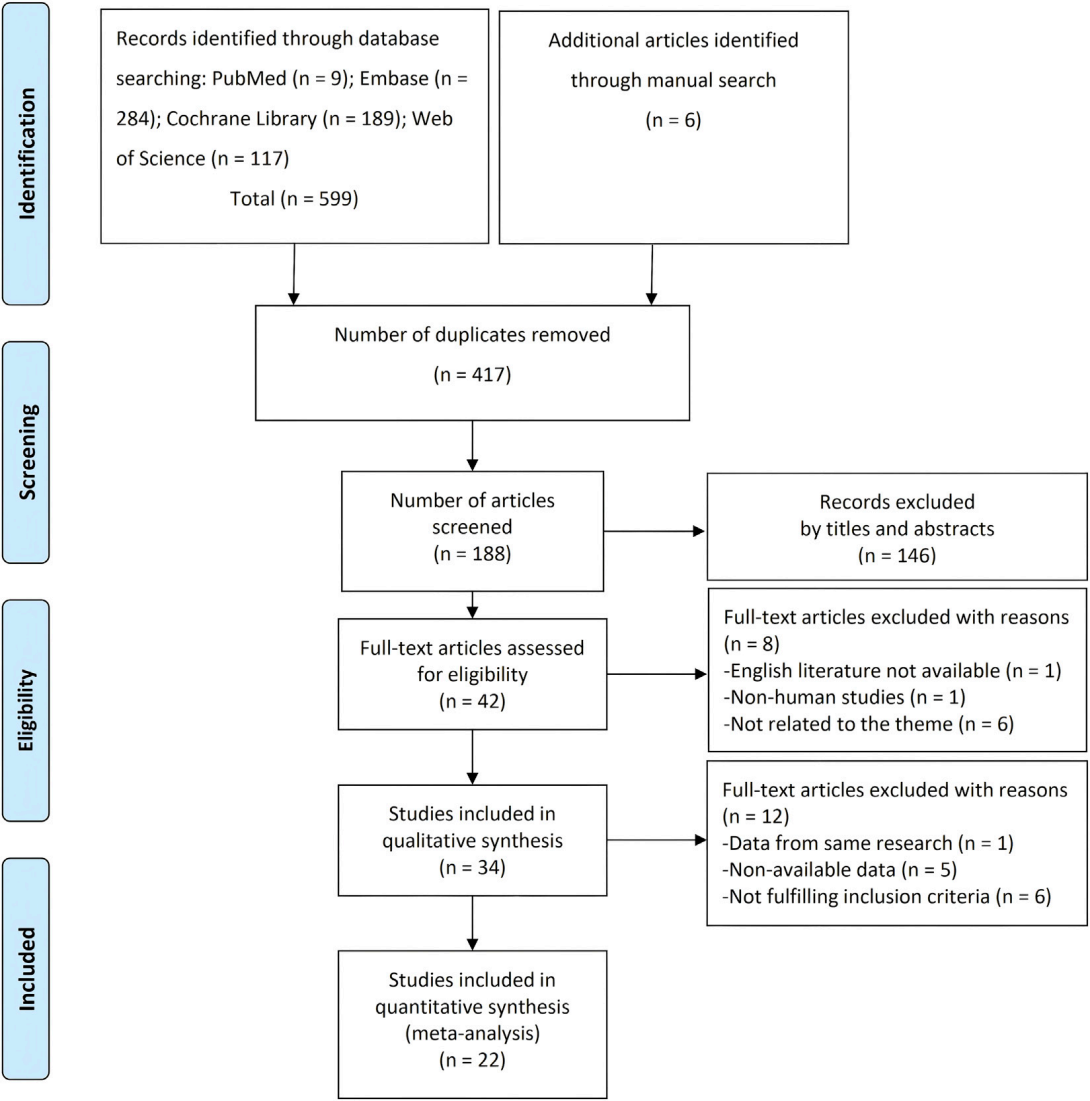
PRISMA flow diagram.

A total of 599 articles were obtained from the database searching, and six additional studies were identified by references. After duplicate removal, 188 records were reviewed. After screening titles and abstracts, 146 records were further excluded. The left 42 articles were assessed by reading the full text. Finally, a total of 34 studies were included in the qualitative synthesis and 22 in the meta-analysis (Booth et al., 1996; Choi et al., 2004; Yokoyama et al., 2007; Bevilacq et al., 2012; Eick et al., 2013; Chitsazi et al., 2014; Deore et al., 2014a; Deore et al., 2014b; El-Sharkawy et al., 2019; El-Sharkawy et al., 2016; Iwasaki et al., 2016; Pradeep et al., 2016; Ramos et al., 2016; Rashidi et al., 2016; Capv et al., 2017; Prakash et al., 2017; AlAhmari et al., 2019; Nędzi-Góra et al., 2020; Zhang et al., 2021; Minić et al., 2022; Talmac et al., 2022; Penala et al., 2016; Pelekos et al., 2019; Keceli et al., 2020; Lecio et al., 2020; Niazi et al., 2020; Qamar et al., 2021; Stańdo et al., 2020; Nguyen et al., 2018; Meghil et al., 2019; Al-Momani, 2021; Gur et al., 2022; Rapone et al., 2022; Sanjay et al., 2025). The 12 excluded studies from the meta-analysis were because of data duplication, non-available data, and not fulfilling inclusion criteria (Figure 1).

### 3.2 Study characteristics

The characteristics of the included studies are summarized in Table 1. Among them, twelve studies were double-blind, placebo-controlled RCT (Deore et al., 2014a; Deore et al., 2014b; El-Sharkawy et al., 2019; Iwasaki et al., 2016; Pradeep et al., 2016; Prakash et al., 2017; Pelekos et al., 2019; Keceli et al., 2020; Lecio et al., 2020; Nguyen et al., 2018; Meghil et al., 2019; Al-Momani, 2021) and 10 studies were placebo-controlled RCT (Booth et al., 1996; Choi et al., 2004; Bevilacq et al., 2012; Eick et al., 2013; Chitsazi et al., 2014; El-Sharkawy et al., 2016; Talmac et al., 2022; Stańdo et al., 2020; Gur et al., 2022; Rapone et al., 2022). Publication years ranged from 1996 to 2022. The sample sizes ranged from 14 participants to 90 participants. The follow-up period was from 4 weeks to 12 months. Treatments include doxycycline (Choi et al., 2004; Lecio et al., 2020), PDT (Chitsazi et al., 2014; Al-Momani, 2021), omega-3 polyunsaturated fatty acids (Deore et al., 2014b; Stańdo et al., 2020), ozone (Rapone et al., 2022), melatonin (El-Sharkawy et al., 2019), propolis (El-Sharkawy et al., 2016), hyaluronic acid (Bevilacq et al., 2012; Eick et al., 2013), herbal medicine (Deore et al., 2014a; Pradeep et al., 2016; Prakash et al., 2017), folic acid (Keceli et al., 2020), vitamin D (Meghil et al., 2019), laser (Talmac et al., 2022; Gur et al., 2022), probiotics (Iwasaki et al., 2016; Pelekos et al., 2019), antibodies against P. gingivalis (Booth et al., 1996; Nguyen et al., 2018). Twenty studies were controlled against SRP alone or SRP with a placebo, and two studies only applied a placebo without SRP (Iwasaki et al., 2016; Prakash et al., 2017). All studies examined PD, and seventeen examined CAL (Choi et al., 2004; Bevilacq et al., 2012; Eick et al., 2013; Chitsazi et al., 2014; Deore et al., 2014a; Deore et al., 2014b; El-Sharkawy et al., 2019; El-Sharkawy et al., 2016; Pradeep et al., 2016; Talmac et al., 2022; Pelekos et al., 2019; Keceli et al., 2020; Lecio et al., 2020; Stańdo et al., 2020; Al-Momani, 2021; Gur et al., 2022; Rapone et al., 2022), eleven examined BOP (Choi et al., 2004; El-Sharkawy et al., 2019; Iwasaki et al., 2016; Prakash et al., 2017; Talmac et al., 2022; Pelekos et al., 2019; Lecio et al., 2020; Stańdo et al., 2020; Nguyen et al., 2018; Al-Momani, 2021; Rapone et al., 2022). Thirteen studies reported the immune outcomes (Choi et al., 2004; Deore et al., 2014a; Deore et al., 2014b; El-Sharkawy et al., 2019; Prakash et al., 2017; Talmac et al., 2022; Keceli et al., 2020; Lecio et al., 2020; Meghil et al., 2019; Al-Momani, 2021; Gur et al., 2022). No adverse events occurred in all the included studies. Most studies demonstrated that the clinical parameters were notably improved in both groups, while immunotherapy offers a more significant clinical benefit compared to placebo. Nevertheless, some studies suggested that adjunctive immunotherapies only brought similar benefits relative to placebo (Booth et al., 1996; Chitsazi et al., 2014; Pelekos et al., 2019; Meghil et al., 2019).

**TABLE 1:** The main characteristics of clinical studies related to immunotherapy of periodontital disease.

Authors and year	Country	Study characteristics	Age mean ± SD (age range) years; sex	Periodontal disease	Negative control group	Test group	Clinical parameters	Biochemical parameters	Follow-up
Chitsazi et al. (2014)	Iran	RCT, SM	Mean age of 29; 15 f, 9 m	Aggressive periodontitis	SRP	SRP+PDT	None of the periodontal parameters exhibited significant differences	NA	3 months
Stańdo M et al. (2020)	Poland	RCT	Mean age 48.4 ± 10.59; 21 f, 19 m	Stage III and IV periodontitis	SRP only	SRP supplemented with 2.6 g of EPA and 1.8 g of DHA (SRP Plus Fish Oil)	Significant improvement of clinical parameters	The salivary levels of pro-inflammatory cytokines/chemokines interleukin (IL)-8 and IL-17 were markedly lower, while the level of anti-inflammatory IL-10 was significantly higher	3 months
Rapone et al. (2022)	Albania	RCT	A mean age of 51.56 ± 10.35; 90 patients, not specified gender	Moderate or severe generalized periodontitis	SRP	SRP + OZONE	Significant differences in periodontal parameters	NA	3 and 6 months
Lecio et al. (2020)	Brazil	RCT, parallel, double-blind	40 patients, gender and age not specified	CP	PLAC —local application of placebo poly lactic-co-glycolic acid (PLGA) nanospheres	DOXY —local application of doxycycline-loaded nanospheres	Significant differences in periodontal parameters	DOXY group exhibited a significant increase in the levels of anti-inflammatory interleukin (IL)-10 and a reduction in the levels of pro-inflammatory cytokines (IL-17, IL-6, INF-y, TNF-α, IL-8) and MMP-9	baseline, and 1, 3, and 6 months
El-Sharkawy et al. (2019), El-Sharkawy et al. (2016)	Egypt	RCT, double-blinded parallel	70 patients, gender and age not specified	Moderate to severe CP	Placebo + SRP group	Melatonin + SRP group	Significant differences in periodontal parameters	Salivary TNF-α levels were significantly lower. However, salivary TNF-α levels exhibited no correlation with other clinical variables in both melatonin and placebo groups.	baseline, 3 and 6 months
Choi et al. (2004)	Korea	RCT	Between the ages of 25 and 64 years; 17 m, 15 f	Incipient to moderate CP	SRP + placebo group	SRP + Sub-antimicrobial dose doxycycline group	Clinical improvement	GCF MMP-8 levels; gingival tissue MMP-9, TIMP-1, and IL-6 levels. (the difference was not statistically significant.)	120 d
Eick et al. (2013)	Germany	RCT	Aged 41 to 72 years; 17 m, 17 f	Moderate or severe CP	SRP only	Hyaluronan gels in two molecular weights were additionally applied during the first 2 weeks after SRP	Significant differences in periodontal parameters	NA	6 months
Deore et al. (2014a), Deore et al. (2014b)	India	RCT, double-blind	60 patients, gender and age not specified	Moderate and severe CP: (defined using the center for disease control Centers for Disease Control and Prevention 2007 criteria)	SRP and a placebo	SRP followed by dietary supplementation of Septilin (herbal immunomodulator drugs) for 3 weeks	Significant differences in periodontal parameters	A significant reduction in the serum CRP level after treatment. There was no significant difference postoperatively between the test group and the control group for the serum CRP levels	3 weeks and 6 weeks
Deore et al. (2014a), Deore et al. (2014b)	India	RCT, double-blind	Between the age of 30 to 60 years; 60 patients, No gender data	Moderate and severe CP [defined by the 2007 criteria]	SRP and a placebo	SRP and dietary supplementation of ω-3 fatty acids. (one 300 mg tablet daily for 12 weeks)	significant differences in periodontal parameters	significant reduction in serum CRP levels after treatment in both groups. no statistically significant changes in serum CRP levels were found	6 weeks and 12 weeks
Bevilacqu et al. (2012)	Italy	RCT, SM	Mean age was 51 years SD ± 9.8; 7 m, 4 f	Moderate-severe CP	Ultrasonic debridement +placebo gel	Ultrasonic mechanical instrumentation associated with 0, 5 ml of amino acids and sodium hyaluronate gel	Significant differences in periodontal parameters	Levels of calprotectin and myeloperoxidase activity	90 days
Keceli et al. (2020)	Turkey	RCT, double-blind, single-centred	Aged 31–61 years; 60 patients, No gender data	Stage II-III periodontitis	SRP + placebo	SRP + folic acid	Significant differences in periodontal parameters	C-reactive protein (CRP) and homocysteine (Hcy)	6 months
Al-Momani (2021)	Saudi Arabia	RCT, SM, parallel arm, doubleblind	The mean age of the 51 patients was 44.7 ± 7.4 years; No gender data	CP	RSD	ICG-aPDT/RSD	Significant differences in periodontal parameters	Interleukin (IL)-17 and interferon (IFN)-γ. serum c-reactive protein (CRP)	6 months
Gur et al. (2021)	Turkey	RCT, single-center	Mean age, 42.33 ± 5.96 years; 23 f, 17 m	Patients with Stage III periodontitis	Only SRP	SRP + diode laser (L) (0.80W power, 940 nm wavelength and 0.80J/s energy level)	Significant differences in periodontal parameters	The gingival crevicular fluid (GCF) levels of interleukin (IL)-17, IL-10, tumor necrosis factor-related weak inducer of apoptosis (TWEAK)	3 months
Pradeep et al. (2016)	India	RCT, single-centre, longitudinal, triple masked, interventional	Aged 25 to 45 years (mean ± SD = 34.76 ± 5.15 years); 33 f, 27 m	CP with moderate to deep pockets	SRP + placebo	SRP+ Aloe Vera Gel	Significant differences in periodontal parameters	NA	6 months
Nguyen et al. (2018)	Vietnam	RCT, two-group, parallel, controlled, double-blind	The mean age of the 60 patients was 37.3 years. There were no statistically significant differences between the two groups on age and gender	CP	SRP followed by a daily use of lozenges containing a sham-immune IgY (placebo)	SRP followed by a daily use of lozenges containing specific IgY against P.gingivalisgingipains	Significant clinical benefits	NA	8 weeks
Talmac et al. (2021)	Turkey	RCT, SM	They were all aged between 18 and 35 years with an average of 31.23 ± 7.4 years; 12 f, 14 m	Generalized aggressive periodontitis in the “Stage III and IV, Generalized, Grade C” group according to “Classification of Periodontal and Peri-Implant Diseases and Conditions 2017”	Only SRP	G1:SRP + Er, Cr:YSGG laser group (SRP + Er, Cr:YSGG); G2:SRP + diode laser (940 ± 15 nm) group (SRP + diode)	Improvement of clinical periodontal parameters	There were positive correlations between the reduction of clinical periodontal indices (GI, PI, PD, CAL, and BOP) and the reduction of post-treatment TNF-α, IL-1β, and IL-8 levels in comparison to pre-treatment levels	3 months
El-Sharkawy et al. (2016)	Egypt	RCT	50 patients, gender and age not specified	Moderate to severe CP according to Armitage criteria	Placebo+SRP	Propolis+SRP	The propolis group showed significant greater PD reduction and CAL gain compared to the control group after 3 and 6 months	NA	6 months
Booth et al. (1996)	United Kingdom	RCT	14 patients, aged 25 to 55 years	Patients had at least 20 standing teeth and had already undergone initial periodontal therapy involving oral hygiene instruction and scaling but still had at least two probing pockets with a depth of 5 mm or more which bled after probing	Saline+SRP	MAb to P. gingivalis +SRP	No significant difference in any clinical periodontal indices between the immunized and control patients	NA	12 months
Meghil et al. (2019)	USA	RCT, double‐blind	23 patients, age of 18 years old and above	Moderate to severe periodontitis (Armitage, 1999)	SRP+placebo	SRP+ vitamin D	No significant differences in most of the periodontal parameters in patients involved in this study at the time points tested	Serum vitamin D and for the salivary immune cytokines including CCL‐20, TNF‐α EFABP (epidermal fatty acid‐binding protein), GM‐CSF (Granulocyte-macrophage colony‐stimulating factor), IL‐1β, IL‐2, IL‐4 IL‐5, IL‐6, IL‐8, and IL‐10. The autophagy‐related protein levels were analyzed in PBMCs (peripheral blood mononuclear cells)	16 weeks
Pelekos et al. (2019)	Hong Kong	A double‐blind, paralleled‐arm, RCT	Age (years) :53.3 ± 9.6 (55.0); 26 f, 15 m	CP (Armitage, 1999)	Placebo lozenges+NSPT	Probiotics L. reuteri lozenges+NSPT	No additional clinical effectiveness	NA	180 days
Prakash et al. (2017)	India	RCT, single-centre, controlled, parallel group, double-blind	Aged 18-35 years; 84 patients, gender not specified	Gingivitis	Placebo gel (PG) without any active ingredient	10% Pomegranate extract gel (PEG)	Significant differences in periodontal parameters	PEG showed significantly less increase in IL-1β, IL-8	Following 14, 30 and 60 days
Iwasaki et al. (2016)	Japan	RCT, double-blind, placebo-controlled	Aged 18-36 years; 24 f, 15 m	CP	Placebo capsule	Receive a capsule containing 10 mg of HK L-137	These clinical findings suggest that daily HK L-137 intake can decrease the depth of periodontal pockets in patients undergoing supportive periodontal therapy	NA	12 weeks

Abbreviation: BOP, bleeding on probing; CAL, clinical attachment level; CP, chronic periodontitis; f, female participant; GCF, gingival crevicular fluid; GI, gingival index; NA, not available; NSPT, non-surgical periodontal treatment; m, male participants; PDT, photodynamic therapy; PI, plaque index; PD, probing pocket depth; RCT, randomized controlled trial; RSD, root surface debridement; SM, split-mouth; SRP, scaling and root planing.

### 3.3 Risk of bias

**FIGURE 2:**
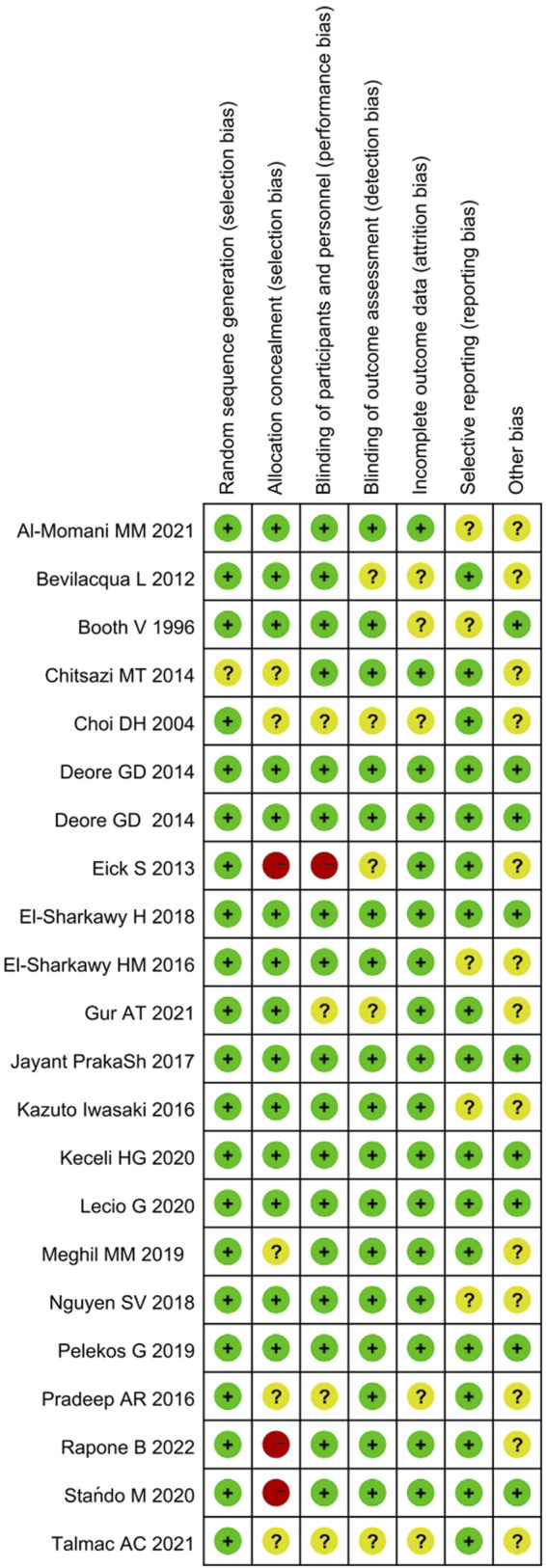
Summary of risk of bias analysis: review authors’ judgment about the different domains for each included study. A green circle (+) indicates a low risk of bias, a yellow circle (?) an unclear risk of bias, and a red circle (−) a high risk of bias in the respective domain.

The RoB rating for each study is presented in Figure 2. Overall, 3 studies were classified with high level of RoB due to the absent description of allocation concealment, 12 with unclear RoB because of insufficient method description, and 7 studies were ranked as low RoB (Supplementary Figure S1).

### 3.4 Effectiveness

#### 3.4.1 Periodontal pocket depth

**FIGURE 3:**
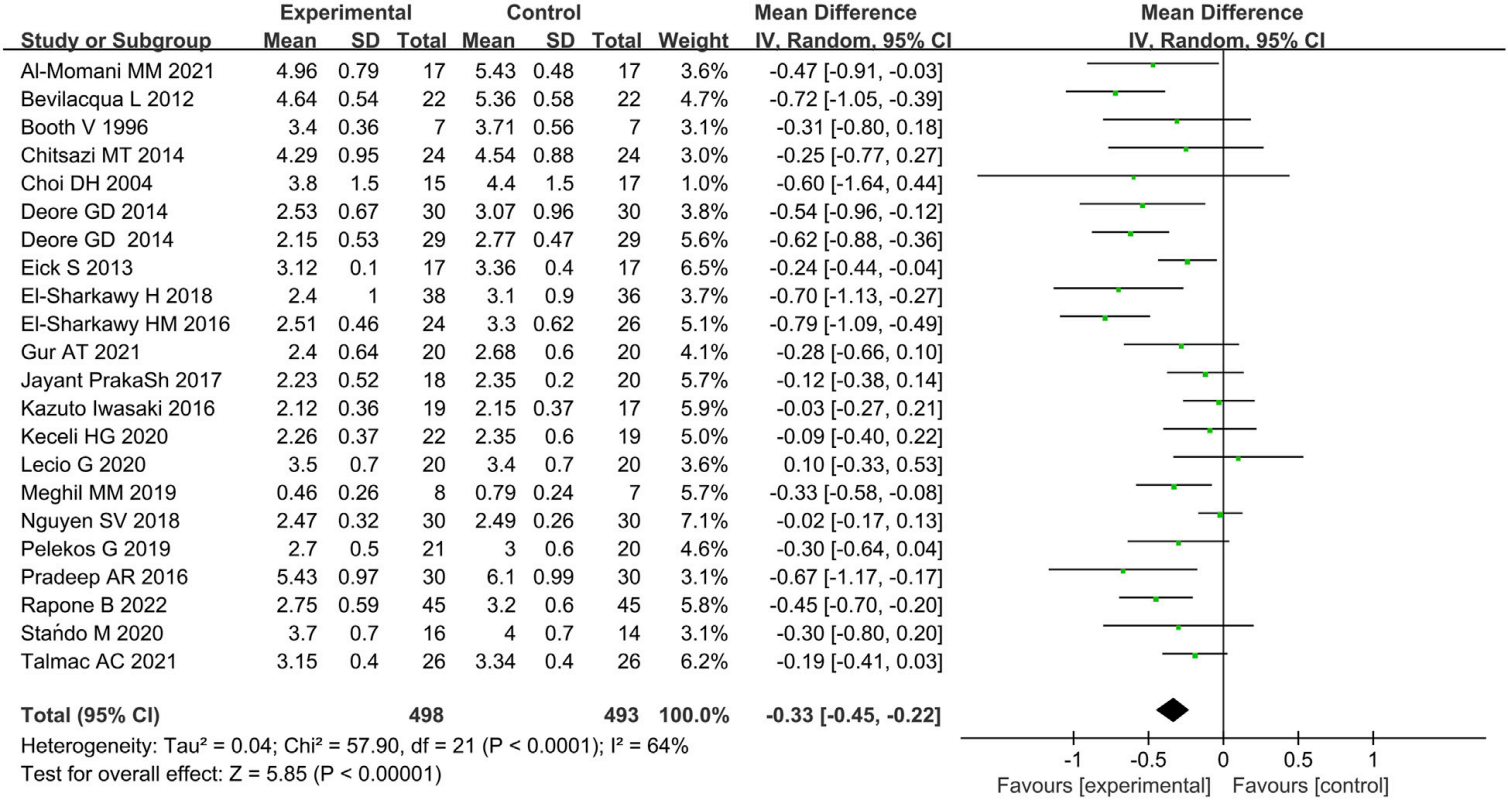
Forest plot of overall PD reduction at 3 months follow-up. WMD, weighted mean difference; CI = confidence interval.

**FIGURE 4:**
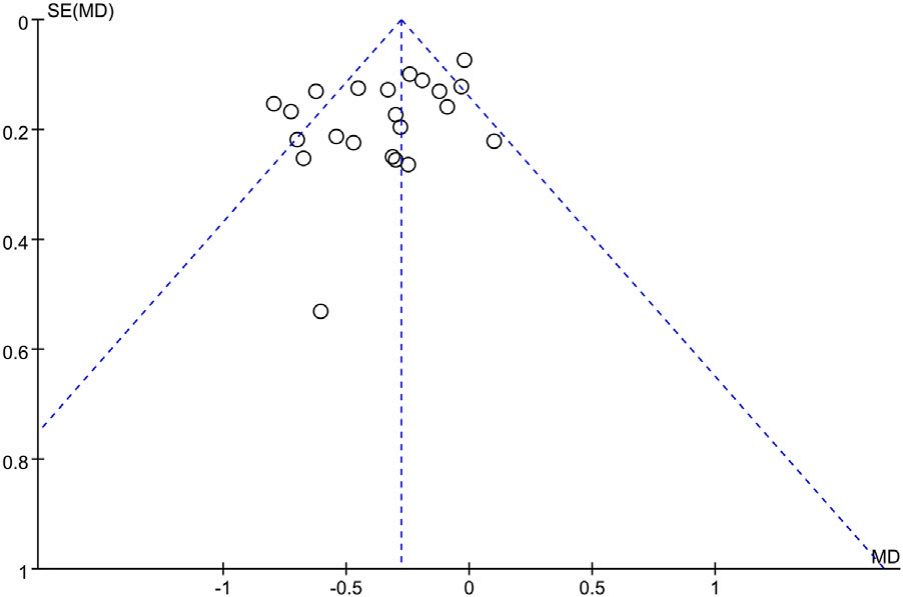
Funnel plot of overall PD reduction at 3 months follow-up.

**FIGURE 5:**
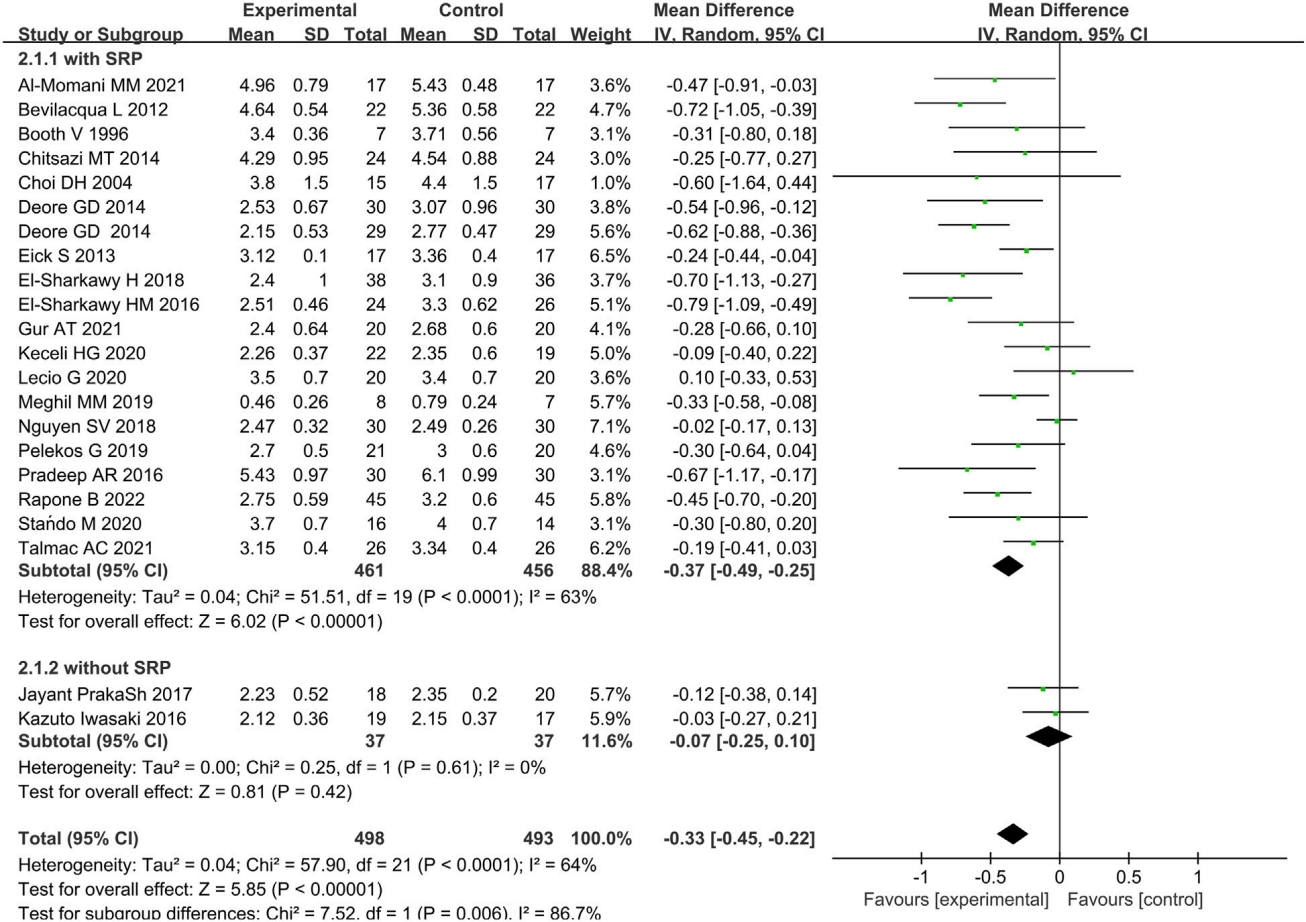
Forest plot of between the immunotherapy group and the non-immunotherapy group subgrouped according to the presence or absence of SRP (immunotherapy + SRP or immunotherapy vs. no immunotherapy).

Twenty-two studies provided data on the efficacy of immunotherapy on our primary outcome (PD reduction) at 3 months. Substantial statistical heterogeneity across the included studies was identified (I^2^ = 64%). Meta-analysis showed that the reduction of PD was associated with immunotherapies when compared with placebo therapy (95% CI = −0.45 to 0.22 mm,χ^2^ = 57.90) (Figure 3). There was no significant publication bias (Figure 4). Subgroup analyses were thus performed to assess whether the combination of immunotherapies with SRP lead to a difference in results compared to placebo treatment (Iwasaki et al., 2016; Prakash et al., 2017). The result reflected that the control groups shown significantly higher PD compared to the immunotherapies with SRP (MD – 0.37 [−0.49, −0.25],p < 0.00001, 95% CI), as well as compared to immunotherapies alone (MD − 0.07 [−0.25, 0.10], p = 0.42, 95% CI). In addition, the immunotherapies combined with SRP could produce a significantly greater effect (lower PD) relative to the undergoing immunotherapies only without SRP (p = 0.006; I^2^ = 86.7%) (Figure 5).

#### 3.4.2 Clinical attachment level

**FIGURE 6:**
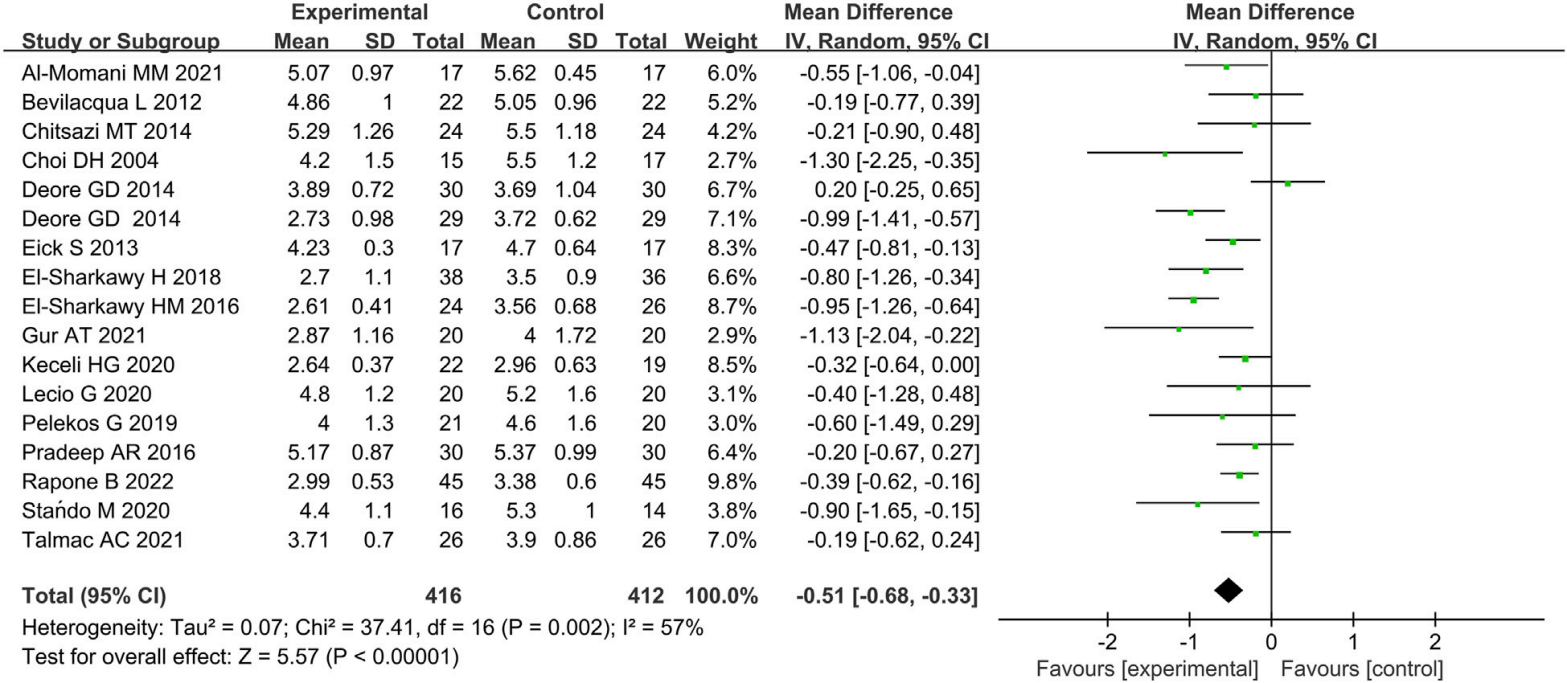
Forest plot of CAL gain at 3 months. WMD, weighted mean difference; CI = confidence interval.

Seventeen studies included data on the effects of immunotherapies on CAL at 3 months (Choi et al., 2004; Bevilacq et al., 2012; Eick et al., 2013; Chitsazi et al., 2014; Deore et al., 2014a; Deore et al., 2014b; El-Sharkawy et al., 2019; El-Sharkawy et al., 2016; Pradeep et al., 2016; Talmac et al., 2022; Pelekos et al., 2019; Keceli et al., 2020; Lecio et al., 2020; Stańdo et al., 2020; Al-Momani, 2021; Gur et al., 2022; Rapone et al., 2022). Meta-analysis demonstrated that immunotherapies with SRP were associated with significantly improved CAL value when compared with placebo with considerable heterogeneity (p < 0.00001, χ^2^ = 37.41, I^2^ = 57%) (Figure 6; Supplementary Figure S2).

#### 3.4.3 BOP

**FIGURE 7:**
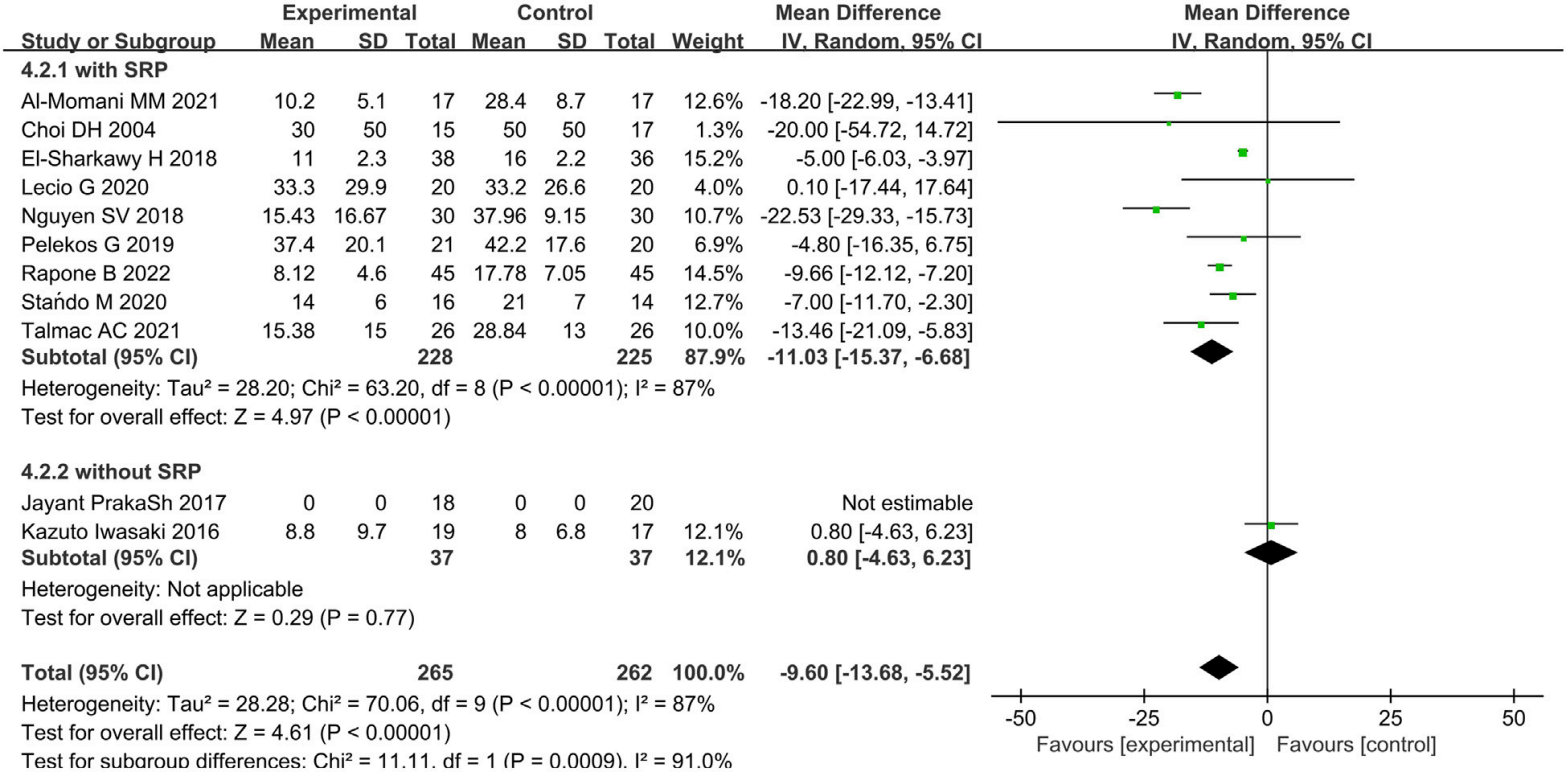
Forest plot of BOP (%) between the immunotherapy group and the non-immunotherapy group subgrouped according to the presence or absence of SRP (immunotherapy + SRP or immunotherapy versus no immunotherapy). CI = confidence interval.

In terms of BOP, eleven articles were analyzed (Choi et al., 2004; El-Sharkawy et al., 2019; Iwasaki et al., 2016; Prakash et al., 2017; Talmac et al., 2022; Pelekos et al., 2019; Lecio et al., 2020; Stańdo et al., 2020; Nguyen et al., 2018; Al-Momani, 2021; Rapone et al., 2022). The results presented WMD of −11.03% (95% CI = −15.37% to −6.68%, p < 0.00001, nine studies (Choi et al., 2004; El-Sharkawy et al., 2019; Talmac et al., 2022; Pelekos et al., 2019; Lecio et al., 2020; Stańdo et al., 2020; Nguyen et al., 2018; Al-Momani, 2021; Rapone et al., 2022)), 0.80% (95% CI = −4.63%–6.23%, p = 0.77, two study (Iwasaki et al., 2016; Prakash et al., 2017)), and −9.60% (95% CI = −13.68% to −5.52%, p < 0.00001) for immunomodulatory therapy with SRP, immunomodulatory therapy only, and overall comparison, respectively. Statistical significance was found, favoring the periodontal immunomodulatory therapy treatment group (Figure 7; Supplementary Figure S3). However, the comparison demonstrated a high heterogeneity for overall comparison (p < 0.00001, I^2^ = 87%).

### 3.5 Immunomodulator

**FIGURE 8:**
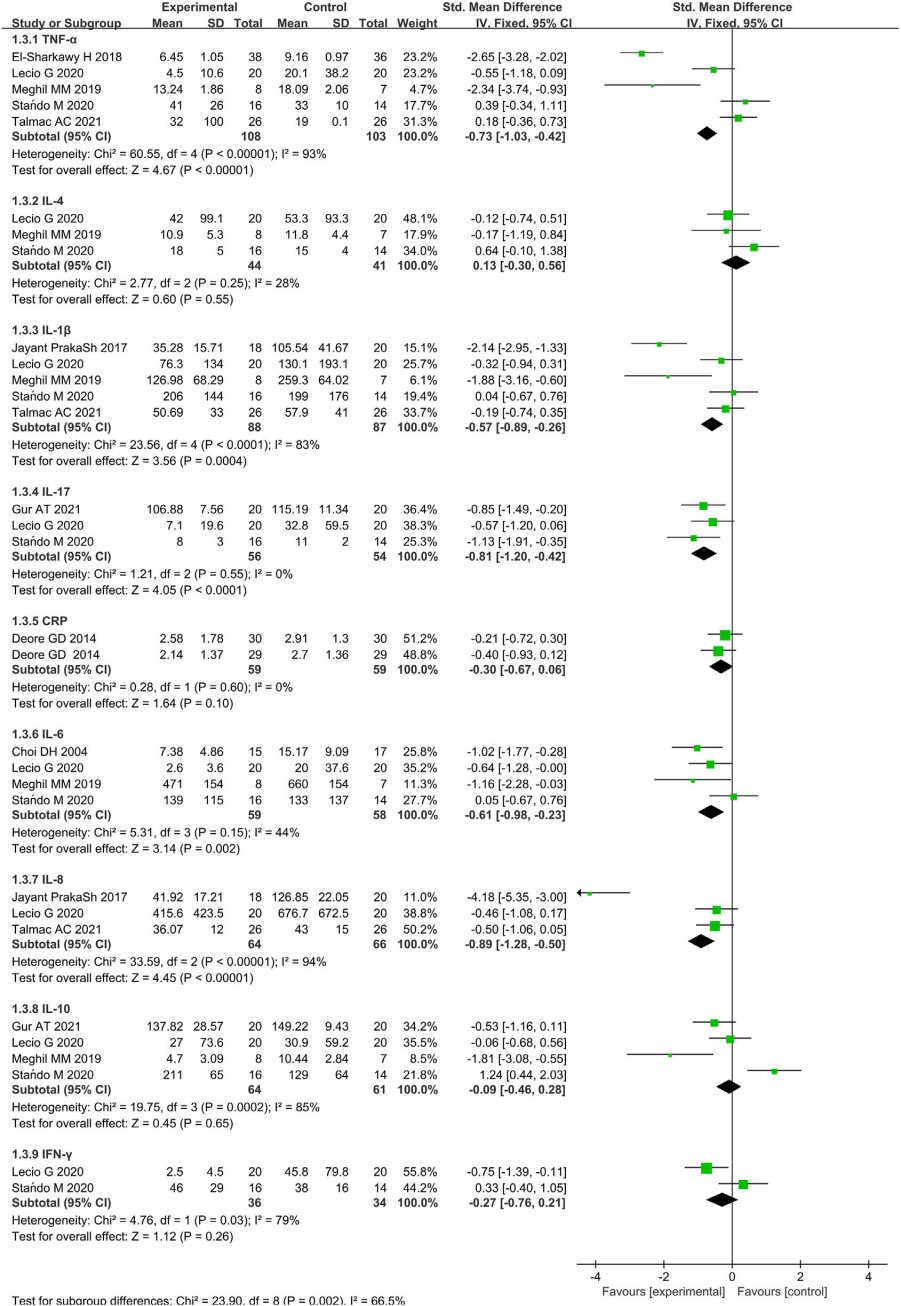
Forest plot of immunological parameters (pg/mL) at 3 months follow-up. CI = confidence interval.

Thirteen studies reported results regarding immunological parameters of the outcomes. Sometimes these parameters were reported as totals instead of concentrations, sometimes they were only provided in graphical form, which impeded direct comparison of these results. Additionally, time points of analysis were inconsistent among all the included studies, which further reduce the power of meta-analysis. In general, ten studies reporting results of immunological parameters were quantitatively analyzed (Figure 8).

#### 3.5.1 Studies of IL-1β,IL-17,IL-6,IL-8,and TNF-α

TNF-α was investigated across 5 studies (El-Sharkawy et al., 2019; Talmac et al., 2022; Lecio et al., 2020; Stańdo et al., 2020; Meghil et al., 2019) (108 cases and 103 controls). Participants received immunotherapy had lower concentrations of TNF-α compared to the control (SMD = −0.73, 95% CI [-1.03, −0.42], p < 0.00001). The variability in differences regarding TNF-α levels was also significant (Q-value = 60.55; p < 0.00001; and I^2^ = 93%).

IL-1β measurements were extracted from 5 studies (Prakash et al., 2017; Talmac et al., 2022; Lecio et al., 2020; Stańdo et al., 2020; Meghil et al., 2019) (88 cases and 87 controls). IL-1β levels reduced significantly more when immunotherapies rather than placebo were combined with SRP (SMD = −0.57, 95% CI [-0.89, −0.26], p = 0.0004). The heterogeneity was large (I^2^ = 83%). Heterogeneity is higher in studies that measured this immune mediator because of different types of samples, sampling methods and relative assays (GCF or saliva).

IL-17 measurements were extracted from 3 studies (Lecio et al., 2020; Stańdo et al., 2020; Gur et al., 2022) (56 cases and 54 controls). IL-17 levels were significantly reduced in the SRP with immunotherapy participants at 3 months after treatment (p < 0.0001). The heterogeneity in IL-17 between studies was not significant (Q-value = 1.21; p = 0.55; and I^2^ = 0%).

IL-6 measurements were extracted from 4 studies (Choi et al., 2004; Lecio et al., 2020; Stańdo et al., 2020; Meghil et al., 2019) (59 cases and 58 controls). IL-6 levels were significantly lower in the test group compared to the control (p = 0.002). However, the heterogeneity of IL-6 among the studies was not significant as IL-17 (Q-value = 5.31; p = 0.15; and I^2^ = 44%).

IL-8 measurements were extracted from 3 studies (Prakash et al., 2017; Talmac et al., 2022; Lecio et al., 2020) (64 cases and 66 controls). The GCF concentrations of IL-8 were significantly lower in the test group compared to the control group (p < 0.00001). The variability in differences in IL-8 levels was significant (Q-value = 33.59; p < 0.00001; and I^2^ = 94%). The quantitative measures of included studies also emerged as a significant moderator as TNF-α.

#### 3.5.2 Studies of IL-4, 10, CRP, and IFN-γ

The meta-analysis showed no significant difference in levels of IL-4 between test and control groups across 3 included studies (Lecio et al., 2020; Stańdo et al., 2020; Meghil et al., 2019). (SMD 1.80 [−0.89, 4.49], p = 0.55, 95% CI). The heterogeneity in levels of IL-4 between studies was not significant (Q-value = 2.77; p = 0.25; and I^2^ = 28%).

Results from 4 studies (Lecio et al., 2020; Stańdo et al., 2020; Meghil et al., 2019; Gur et al., 2022) (test/control = 64/61) measured IL-10 levels. No significant difference was noticed between the test and control groups for the expression of IL-10 (p = 0.65).

Identically, the levels of IFN-γ did not show statistical difference between the test and control groups across the 2 included studies (Lecio et al., 2020; Stańdo et al., 2020) (p = 0.26). The heterogeneity in levels of IFN-γ between studies was, however, significant (Q-value = 4.76; p = 0.03; and I^2^ = 79%).

Results from 2 studies (Deore et al., 2014a; Deore et al., 2014b) (59 cases and 59 controls) measured serum C-reactive protein (CRP) levels were also included in the meta-analysis. We used the fix-effects model, compared to controls, finding no significant difference in serum CRP levels in the test groups (SMD = −30, 95% CI [-0.67,0.06], p = 0.10). The heterogeneity in serum CRP levels between studies was also not significant (Q-value = 0.28; p = 0.60; and I^2^ = 0%).

#### 3.5.3 Other mediators

Levels of IL-2, IL-5, IL-11, IL-12, IL-32, CXCL8, CCL-20, and transforming growth factor-β (TGF-β) were also measured in studies (Stańdo et al., 2020; Meghil et al., 2019; Gürkan et al., 2005; Gürkan et al., 2008). The meta-analysis could not be performed due to the limited number of studies.

## 4 Discussion

Periodontitis is the second cause of tooth loss worldwide (Ba et al., 2000). As an inflammatory disease, the main culprit identified is the bacterial biofilm growing on the tooth surfaces (Potempa et al., 2000). There are plenty of therapeutic approaches for periodontitis in clinic, among which SRP has been recognized as the gold standard for the treatment of periodontitis for decades (Aljateeli et al., 2014). This is because SRP focuses on the removal of pathogenic plaque and contributes to a chance to re-establish the metabolic balance between the environment (periodontal microbes) and host (local tissue). However, SRP is not always effective because the treated sites might be recolonized with the microbiota right away due to the breakdown of the previous healthy periodontal metabolic balance (Mombelli, 2000). This situation tends to occur at sites of deep periodontal pocket, which is one of the most challenging issues for clinicians (Graziani et al., 2019). Therefore, immunotherapies have been proposed in the treatment of periodontitis to re-establish the periodontal homestasis and modulate the repairing capability of host tissue (Yang et al., 2021). In this study, we included 22 clinical trials concerning immunomodulatory therapy for periodontal disease, including antibiotics, essential oils, laser and photodynamic therapy, probiotics, etc. Using intervention factors, the clinical manifestations of periodontal disease in clinical cases were discussed to infer the therapeutic effect. Some of these cases were also measured by local or systemic immune regulators, and changes in cytokines before and after the intervention were demonstrated (Choi et al., 2004; Deore et al., 2014a; Deore et al., 2014b; El-Sharkawy et al., 2019; Prakash et al., 2017; Talmac et al., 2022; Keceli et al., 2020; Lecio et al., 2020; Meghil et al., 2019; Al-Momani, 2021; Gur et al., 2022). We first analyzed the effect of immunomodulatory factors in the intervention treatment and further explored the immunomodulatory factors that may influence the treatment prognosis after periodontal intervention.

Many studies have found that periodontal disease can reverse the immune regulatory factors in the local and even systemic microenvironment after intervention treatment (Bokhari et al., 2012; Türer et al., 2017; D'Aiuto et al., 2018; Albuquerque-Souza et al., 2019). Li et al. reported biomimetic immunomodulation by crosstalk with nanoparticulate regulatory T cells in animal models of periodontitis, which inhibited the proliferation and activation of T cells, reduced secreting of TNF-α, IFN-γ, and IL-17a, suppressed excessive immune responses, alleviated inflammation and curbed alveolar bone resorption (Sun et al., 2023; Hienz et al., 2015). Preclinical studies also found resolvin E1 turned over bone loss, reversed inflammatory gene expression, and significantly reduced osteoclast density, inflammatory cell infiltration, and systemic CRP and IL-1β levels (Hasturk et al., 2007; Lee et al., 2016). The *in vivo* data from animal studies highlighted the potential mechanism for the improved efficacy of the immunomodulation in the treatment of periodontitis. Our study found that compared with the control group without intervention or with only periodontal scaling, the combination of SRP and immunomodulatory therapy showed a general improvement in PD, CAL, BOP, and more importantly, in the experimental group, pro-inflammatory factors (IL-1β, IL-6, IL-8, IL-17, and TNF-α) were notably reduced. The results of human trials are consistent with data from animal studies with immunotherapy.

To further clarify the role of immune intervention therapy in periodontitis, we conducted a further analysis on the effect of immunotherapy, and found that immunomodulatory treatment alone reduced PD compared with blank control group, but BOP did not respond obviously. However, conclusions regarding the comparison of ‘immunotherapy combined with SRP’ versus ‘immunotherapy alone’ should be interpreted with caution, as this subgroup analysis was based on a small sample size (only two included studies), necessitating careful extrapolation of these findings. However, the combination of immunomodulatory therapy and SRP can achieve good clinical outcomes, as indicated by PD, BOP, and CAL changes. Minagawa, et al. have proved that, resveratrol suppresses IL-1β, IL-8, and monocyte chemoattractant-1 (MCP-1) in human gingival epithelial cells, which partly explained the reaction of host cells towards immunomodulation treatment (Minagawa et al., 2015). Also, our findings further suggested that the progression of periodontitis may be due to the dysregulation of the periodontal local immune microenvironment.

During the progression of periodontitis, abundant inflammatory factors are stimulated in a spatiotemporal order. These factors trigger a series of pro-inflammatory or anti-inflammatory responses. The combat between protective and destructive responses decides the fate of compromised periodontal tissue. If the pro-inflammatory progress went out of control, the disease would eventually lead to alveolar bone resorption, gum atrophy, and tooth loosening (Cekici et al., 2000; Slots, 2000b; Fu et al., 2016). On the other hand, if these factors could reach a new balance, and contribute to the microenvironment of periodontal tissue, the regeneration progress might be activated (Gonzales et al., 2014; Pan et al., 2019). However, the interpretation of the concentration of a certain factor sometimes has two sides. For example, IL-6 may activate a classical pathway and a trans-signaling pathway, which may have predominantly anti- and proinflammatory activities respectively (Scheller et al., 2011). It has also been suggested that IL-17 contributes to disease progression in early-stage experimental periodontitis in mice, but has a protective role in the later stages (Wilharm et al., 2022). Our findings suggested that alterations in cytokine levels were associated with immunotherapy treatment outcomes in periodontitis. Further investigations are warranted to examine the potentiality of using inflammatory cytokines as real-time, sensitive, and reliable predictive markers for the comprehensive treatment system of periodontitis. It has to be pointed out that immunotherapy also has some drawbacks, especially when been systemically administrated which can cause adverse events (AEs). The unique mechanism of action of immunomodulatory therapy may elicit a toxicity spectrum different from that of traditional therapy, and the risk-benefit ratio needs to be evaluated separately.

A critical limitation of current immunomodulatory approaches is their tendency to target broad-spectrum inflammatory mediators, which may not resolve the complex periodontal inflammatory cascade effectively. Furthermore, the chronic nature of periodontitis requires prolonged systemic administration of some host-modulating therapies (HMTs), raising concerns about potential severe adverse events (AEs), such as increased risk for serious bacterial, fungal, and viral infections, or drug-induced Lupus, reported for anti-cytokine therapies. These safety concerns underscore the necessity to shift from broad-spectrum inhibition toward more precise, localized targets. The wide individual variation in patient susceptibility and response to treatment, influenced by genetics, systemic or environmental factors, further highlights the need for personalized treatment approaches and patient stratification in future studies.

This study is a pioneering and comprehensive meta-analysis (including 22 RCTs, all human trials) to assess the clinical efficacy of immunotherapy in the treatment of periodontitis. The results of the meta-analysis showed that adjunctive immunotherapies with SRP provided a note-worthy clinical benefit in clinical tests (PD, BOP, and CAL) when compared with the control. Meanwhile, immunomodulatory treatment significantly decreased levels of IL-1β,IL-17,IL-6,IL-8, and TNF-α.

This study has several limitations. Firstly, the sample size of the included studies was relatively limited. Large-scale, well-designed RCTs are also required to validate our conclusion and to identify which type of immunotherapy is more effective for different stages of periodontal inflammatory diseases. Follow-up periods across studies ranged from 4 weeks to 12 months, which may impair comparability of clinical outcomes. A major limitation is the high statistical heterogeneity observed across multiple analyses (e.g., PD: *I* ^2^ = 64%, BOP: *I* ^2^ = 87%). This high *I* ^2^ value suggests substantial clinical and methodological diversity among the included trials. In addition, fixed-effects models were used to estimate the Ess of mediators, which may be inaccurate when heterogeneity is large. Further investigations are warranted to elucidate sources of heterogeneity of the results. Also, during the literature selection process, not all existing literature could be screened and some studies did not provide original data, also providing a potential bias source. Finally, restricting the inclusion criteria to English-language manuscripts may introduce language bias. The inclusion of studies assessing different biofluids (e.g., GCF, saliva) also introduces variability that should be acknowledged. Last but not least, the wide range of immunomodulatory therapies included may contribute to imprecision in the findings.

## Data Availability

The datasets presented in this study can be found in online repositories. The names of the repository/repositories and accession number(s) can be found in the article/Supplementary Material.
