# Supplementary figures and images for: Clinical effect of immunomodulatory therapy in periodontitis: a systematic review and meta-analysis
Source: Front Bioeng Biotechnol. 2025 Nov 20;13:1693365. doi: 10.3389/fbioe.2025.1693365 (PMC12675453; doi:10.3389/fbioe.2025.1693365)

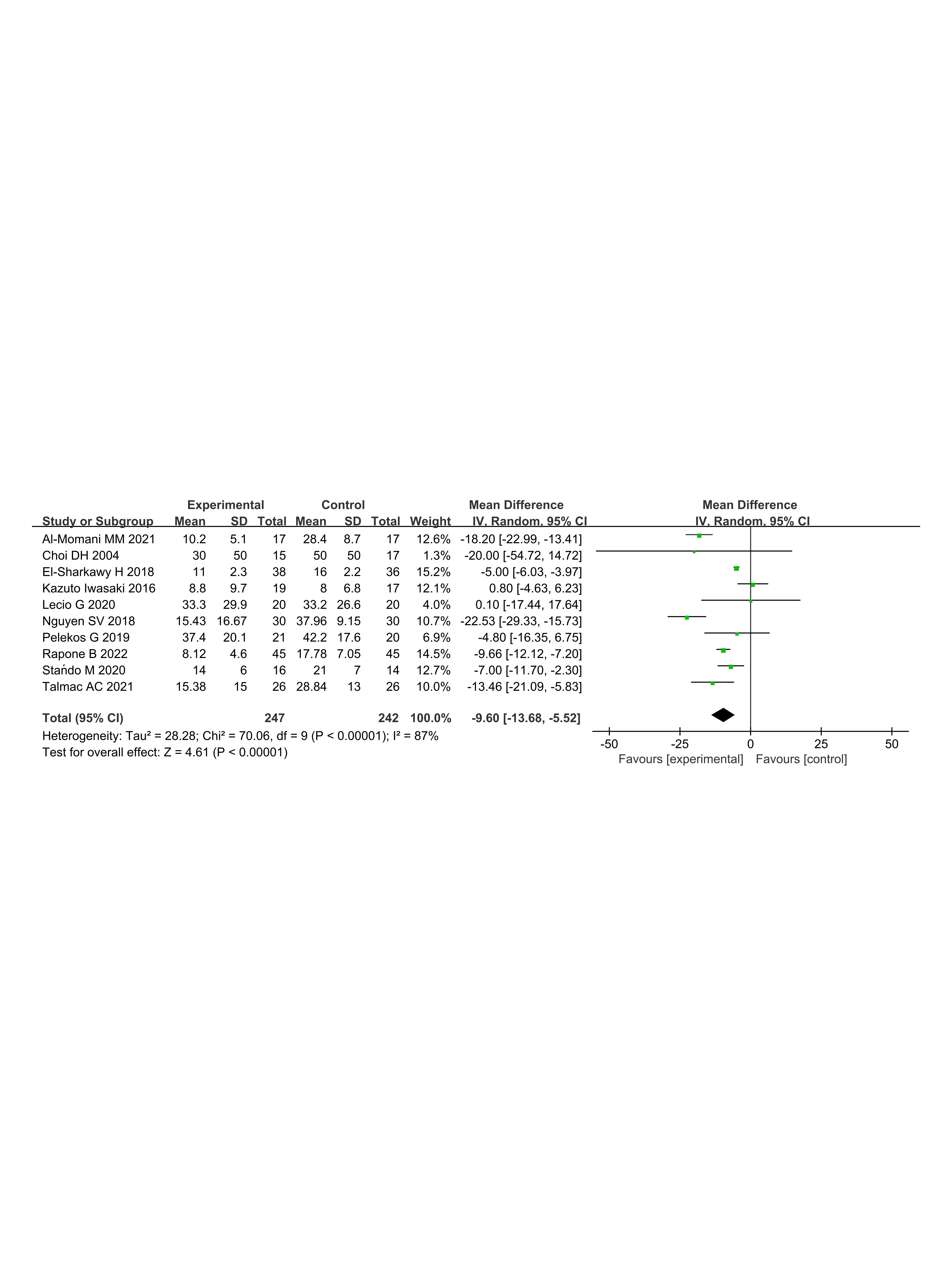

Supplement: Supplementary file 1 [file Image3.jpeg]

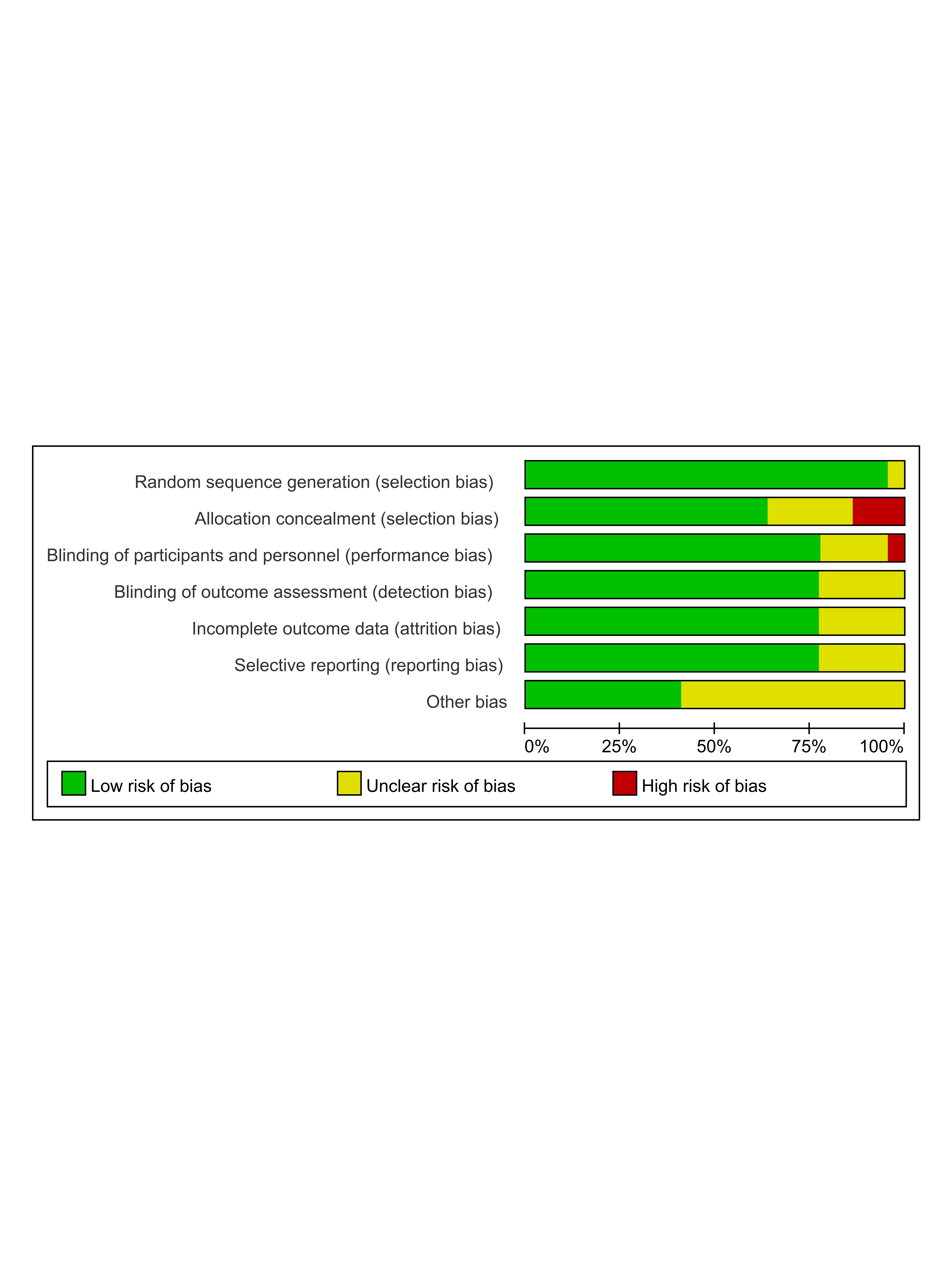

Supplement: Supplementary file 4 [file Image1.jpeg]

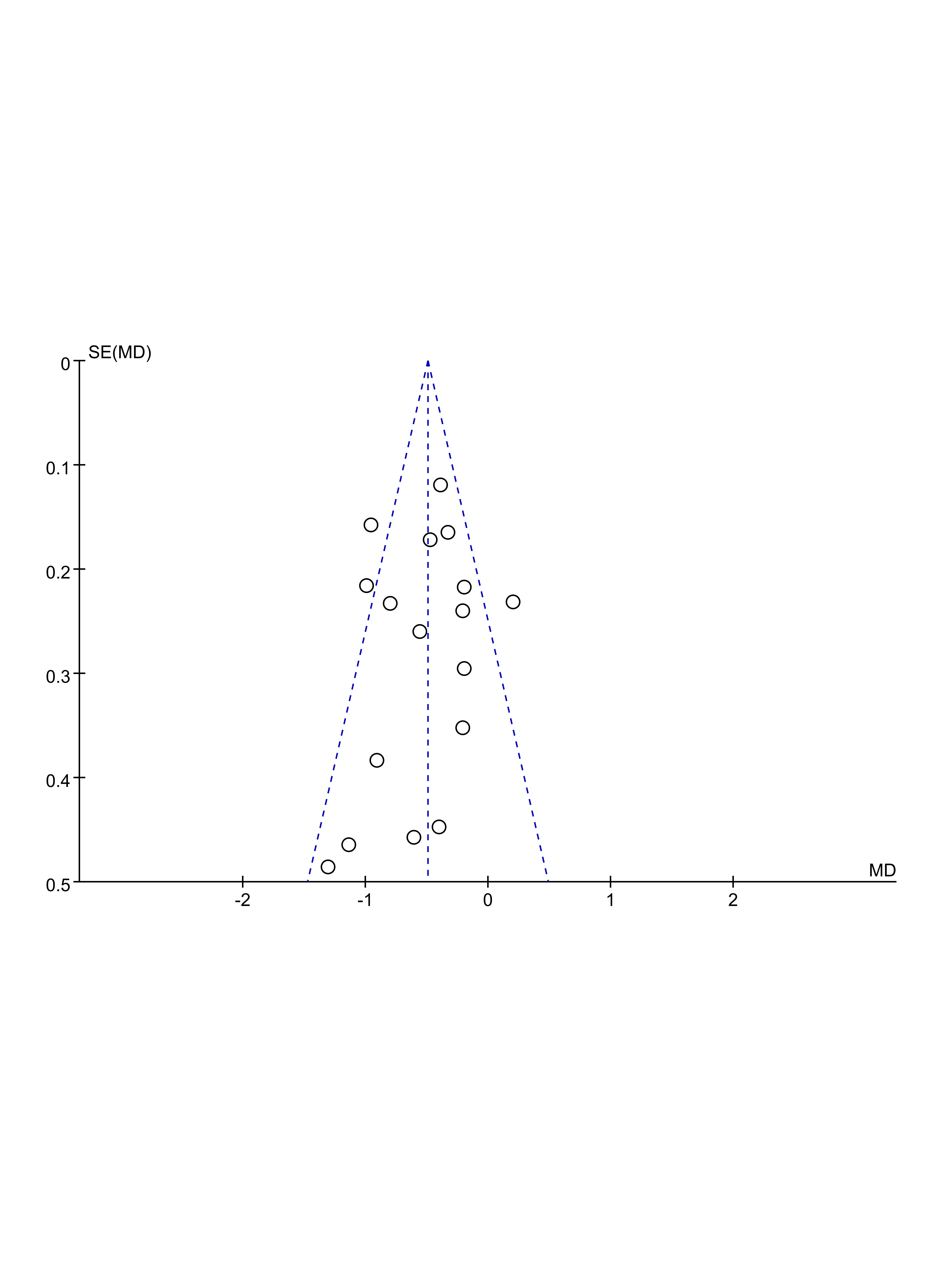

Supplement: Supplementary file 5 [file Image2.jpeg]
