# Supplementary material for: Clinical effect of immunomodulatory therapy in periodontitis: a systematic review and meta-analysis
Source: Front Bioeng Biotechnol. 2025 Nov 20;13:1693365. doi: 10.3389/fbioe.2025.1693365 (PMC12675453; doi:10.3389/fbioe.2025.1693365)
Supplement: Supplementary file 2 [file Supplementaryfile1.docx]

**Supp Figure legends**

Supp Figure 1 Depicts the overall judgment of bias for all 22 included studies according to the seven domains.

Supp Figure 2 Funnel plot of CAL at 3 months follow-up.

Supp Figure 3 Forest plot of BOP (%) at 3 months follow-up. CI = confidence interval.

Table 1. The main characteristics of clinical studies related to immunotherapy of periodontal disease.

# search strategy

Cochrane Library

Search Name: ((Immunotherapy or Immunotherapies)) AND (Periodontitis or Periodontitides or Pericementitis or Pericementitides)

Comment:

ID Search Hits

#1 MeSH descriptor: [Immunotherapy] explode all trees 11645

#2 (immunotherapies): ti,ab,kw OR (immune therapy):ti,ab,kw OR (immunization therapy):ti,ab,kw OR (immunotherapeutic):ti,ab,kw (Word variations have been searched) 35362

#3 #1 or #2 41539

#4 MeSH descriptor: [Periapical Abscess] explode all trees 45

#5 (peridontal disease):ti,ab,kw OR (periodontal disease):ti,ab,kw OR (periodontitis):ti,ab,kw OR (periodontosis):ti,ab,kw OR (periodontopathy):ti,ab,kw (Word variations have been searched) 13839

#6 #4 or #5 13874

#7 #3 and #6 146

PubMed

(("immunotherapy"[MeSH Terms] OR "immunotherapy"[All Fields] OR "immunotherapies"[All Fields] OR "immunotherapy s"[All Fields] OR ("immunotherapy"[MeSH Terms] OR "immunotherapy"[All Fields] OR "immunotherapies"[All Fields] OR "immunotherapy s"[All Fields])) AND ("periodontal"[All Fields] OR "periodontally"[All Fields] OR "periodontically"[All Fields] OR "periodontics"[MeSH Terms] OR "periodontics"[All Fields] OR "periodontic"[All Fields] OR "periodontitis"[MeSH Terms] OR "periodontitis"[All Fields] OR "periodontitides"[All Fields] OR ("periodontal"[All Fields] OR "periodontally"[All Fields] OR "periodontically"[All Fields] OR "periodontics"[MeSH Terms] OR "periodontics"[All Fields] OR "periodontic"[All Fields] OR "periodontitis"[MeSH Terms] OR "periodontitis"[All Fields] OR "periodontitides"[All Fields]) OR ("periodontitis"[MeSH Terms] OR "periodontitis"[All Fields] OR "pericementitis"[All Fields]) OR ("periodontitis"[MeSH Terms] OR "periodontitis"[All Fields]))) AND (clinicaltrial[Filter] OR randomizedcontrolledtrial[Filter])

9

WOS

((((TS=(immunotherapy)) OR TS=(immunotherapies)) OR AB=(immunotherapy)) OR AB=(immunotherapies)) AND ((((((((TS=(periodontitis)) OR TS=(periodontitides)) OR TS=(pericementitis)) OR TS=(pericementitides)) OR AB=(periodontitis)) OR AB=(periodontitides)) OR AB=(pericementitis)) OR AB=(pericementitides))

117

Embase
((immunotherapy): ti, ab, kw OR ((immunotherapy)/exp) OR ((immunotherapies):ti,ab,kw)) AND ((periodontitis)/exp OR ((periodontitis):ti,ab,kw) OR ((periodontitides): ti,ab,kw) OR ((pericementitis):ti,ab,kw) OR ((pericementitides):ti,ab,kw))

284
