# Supplementary material for: Clinical effect of immunomodulatory therapy in periodontitis: a systematic review and meta-analysis
Source: Front Bioeng Biotechnol. 2025 Nov 20;13:1693365. doi: 10.3389/fbioe.2025.1693365 (PMC12675453; doi:10.3389/fbioe.2025.1693365)
Supplement: Supplementary file 3 [file Table1.docx]

The effect of immunotherapy in periodontitis: A meta-analysis

**Yubing Zhang, Xu Qin and**

**Supplementary Table S1.** Reasons for exclusions of studies in full text screening.

| **Study** | **Reason for Exclusion in the Full Text Screening** |
| --- | --- |
| Anwaristi et al., 2020 | wrong study design: no control group |
| Bhalla et al., 2019 | wrong study design: no control group without active  substances |
| Chug et al., 2020 | wrong intervention: CoQ10 administration one month after SRP in patients who did not respond |
| Folkers et al., 1977 | wrong study design: narrative review |
| Hanioka et al., 1994 | wrong intervention: start of CoQ10 administration before SRP |
| Lister et al., 1995 | wrong study design: letter to the editor |
| Manthena et al., 2015 | wrong indication: not clearly periodontitis patients |
| Mathews-Brzozowska et al., 2007 | wrong study design: no control group |
| Matsumura et al., 1973 | wrong intervention: no SRP |
| Nakamura et al., 1973 | no intervention |
| Pitale et al., 2012 | wrong indication: generalized gingivitis or slight periodontitis (1-2 mm CAL) |
| Rasperini et al., 2019 | wrong intervention: combination preparation (micronutrient complex tablets); start of CoQ10  administration before SRP |
| Roopa et al. 2014 | wrong intervention: combination preparations (CNBC gel) |
| Saini et al., 2011 | wrong study design: narrative review |
| Wilkinson et al., 1975 | wrong study design: no control group,  wrong intervention: CoQ10 administration after SRP in patients who were assigned for surgical  correction |
| Wilkinson et al., 1976 | wrong intervention: no SRP |
| Zaki et al., 2012 | wrong intervention: no SRP |

**References**

Anwaristi, A.Y. Effect of coenzyme-q10 in the post-curettage against probing depth, relative attachment loss, and bleeding on probing. J. Syiah Kuala Dent. Soc. 2022, 5, 66–69, https://doi.org/10.24815/jds.v5i2.20015.

Bhalla, A.; Jithendra, K.D.; Shailendra, S. Co-Enzyme Q10:-Another Armour For Novel Periodontal Therapy. 2019.

Chug, A.; Shukla, S. Placement of Sticky Bone™ in patients with generalized periodontitis previously treated with coen-zyme Q10. J. Contemp. Dent. Pract. 2020, 21, 156–160.

Folkers, K.; Watanabe, T. Bioenergetics in clinical medicine-X. Survey of the adjunctive use of coenzyme Q with oral thera-py in treating periodontal disease. J. Med. 1977, 8, 333–348.

Hanioka, T.; Tanaka, M.; Ojima, M.; Shizukuishi, S.; Folkers, K. Effect of topical application of Coenzyme Q10 on adult per-iodontitis. Mol. Asp. Med. 1994, 15, s241–s248, https://doi.org/10.1016/0098-2997(94)90034-5.

Lister, R.E. Coenzyme Q10 and periodontal disease. Br. Dent. J. 1995, 179, 200–201.

Manthena, S.; Rao, M.V.R.; Penubolu, L.P.; Putcha, M.; Harsha, A.V.N.S. Effectiveness of CoQ10 Oral Supplements as an Ad-junct to Scaling and Root Planing in Improving Periodontal Health. J. Clin. Diagn. Res. JCDR 2015, 9, ZC26-8.

Matthews-Brzozowska, T.; Kurhañska-Flisykowska, A.; Wyganowska-Swiatkowska, M.; Stopa, J. Healing of periodontal tissue assisted by coenzyme Q10 with vitamin E—Clinical and laboratory evaluation. Pharm. Rep. 2007, 59 (Suppl. S1), 257–260.

Matsumura, T.; Saji, S.; Nakamura, R.; Folkers, K. Evidence for enhanced treatment of periodontal disease by therapy with coenzyme Q. Int. J. Vitam. Nutr. Res. 1973, 43, 537– 548.

Nakamura, R.; Littaru, G.P.; Folkers, K; Wilkinson, E.G. Deficiency of Coenzyme Q in Gingiva of Patients with Periodontal Disease. J. Int. Vitaminol. Nutr. 1973, 43, 84–92.

Pitale, U.; Khetarpal, S.; Peter, K.; Pal, V.; Verma, E.; Gupta, P. Evaluation of efficacy of coenzyme Q 10 in management of gingivitis & slight periodontitis - a clinical study. Int. J. Curr. Pharm. Res. 2012, 4, 33–38.

Rasperini, G.; Pellegrini, G.; Sugai, J.; Mauro, C.; Fiocchi, S.; Mora, P.C.; Dellavia, C. Effects of food supplements on periodontal status and local and systemic inflammation after nonoperative periodontal treatment. J. Oral Sci. 2019, 61, 213–220, https://doi.org/10.2334/josnusd.18-0048.

Roopa, D.A.; Gupta, R.; Gupta, I.; Chauhan, S.; Pandey, A.; Sharma, N.K. Clinical evaluation of topical application of CNBC gel (Coenzyme Q10) in chronic periodontitis patients. J. Dent. Res. Updates 2014, 1, 13–17.

Saini, R. Coenzyme Q10: The essential nutrient. J. Pharm. Bioallied Sci. 2011, 3, 466–467.

Wilkinson, E.G.; Arnold, R.M.; Folkers, K.; Hansen, I.; Kishi, H. Bioenergetics in clinical medicine. II. Adjunctive treatment with coenzyme Q in periodontal therapy. Res. Commun. Chem. Pathol. Pharmacol. 1975, 12, 111–123.

Wilkinson, E.G.; Arnold, R.M.; Folkers, K. Bioenergetics in clinical medicine. VI. Adjunctive treatment of periodontal disease with coenzyme Q10. Res. Commun. Chem. Pathol.

Pharmacol. 1976, 14, 715–719.

Zaki, N.M. Site-specific delivery of the Nutraceutical COQ10 for periodontal therapy. Int. J. Pharm. Pharm. Sci. 2012, 4, 717–723.
